# Osteoblast differentiation from synovial fluid cells in juvenile idiopathic arthritis (JIA)

**DOI:** 10.1186/1546-0096-10-S1-A115

**Published:** 2012-07-13

**Authors:** Marija Jelusic-Drazic, Elvira Lazic Mosler, Danka Grcevic, Mandica Vidovic, Ana Marusic, Natasa Kovacic

**Affiliations:** 1 University Hospital Center Zagreb, Zagreb, Croatia, Croatia; 2 University of Zagreb School of Medicine, Zagreb, Croatia

## Purpose

Juvenile idiopathic arthritis (JIA) is one of the leading causes of disability in children characterized by destruction of articular cartilage and underlying bone, as well as synovial hyperplasia. Since synovia contains osteoblast progenitors, hyperplastic changes may contribute to joint destruction by inhibition of osteoblast differentiation. Osteoblasts are also immunoregulatory cells and their inhibited differentiation and function may affect joint inflammation. The objective of this report is to explore the osteoblastogenic potential of synovial fluid cells in JIA and to examine whether synovial fluid from children with JIA effects osteoblast differentiation of human bone marrow cells in vitro. Additionaly, we aimed to determine and compare the local and systemic expression of osteoblast related genes in patients with JIA.

## Methods

Blood samples were obtained from 21 children with oligoarticular JIA (oJIA), 21 children with polyarticular JIA (pJIA), and 24 control children. Synovial fluid samples were collected from 21 children with oJIA and 6 children with pJIA. Peripheral blood cells from healthy controls and children with JIA, as well as synovial fluid cells from children with JIA were used to analyze the gene expression of Runx1, Runx2, Runx3, osteoprotegerin (OPG) and receptor activator of nuclear factor kappa-B ligand (RANKL) by quantitative polymerase chain reaction. Osteoblastogenesis from synovial cells was induced in α-MEM medium supplemented with 10% fetal bovine serum, 50 μg/ml ascorbic acid and 5 mmol β-glycerophosphate, and assessed by alkaline phospatase (AP) staining of osteoblast colonies on culture day 21. The human bone marrow cells from healthly donor were cultured for 17 days with 10% synovial fluid from children with JIA. Osteoblast differentiation was compared with non-treated cells, assessed by AP activity.

## Results

Synovial cells from children with oJIA formed more AP positive colonies on culture day 21, in comparison to synovial cells from children with pJIA (784.81±216.79 vs. 257.21±68.13 arbitrary units, p<0.001, t-test). As assessed by AP activity synovial fluid from children both with oJIA and pJIA inhibited osteoblast differentiation of human bone marrow cells (0.059±0.026 in oJIA; 0.068±0.019 in pJIA vs. 0,115 ± 0,023 in control cultures, p<0.05, t-test). Gene expression of Runx1, Runx2, Runx3 and RANKL was higher in synovial fluid of children with pJIA than with oJIA.The expression of osteoblast related genes in peripheral blood mononuclear cells from children with JIA was similar to healthy controls.

## Conclusion

Osteoblast differentiation is locally inhibited in children with JIA, which is particularly pronounced in poliarticular disease. Osteoblast differentiation potential of synovial cells may serve as an important prognostic and therapeutic factor in JIA

## Disclosure

Marija Jelusic-Drazic: None; Elvira Lazic Mosler: None; Danka Grcevic: None; Mandica Vidovic: None; Ana Marusic: None; Natasa Kovacic: None.

